# Formulation of Bilayer Benzydamine HCl Patch Targeted For Gingivitis

**DOI:** 10.1155/2016/7598398

**Published:** 2016-12-29

**Authors:** Piyush Sanghai, Tanaji Nandgude, Sushilkumar Poddar

**Affiliations:** Department of Pharmaceutics, Dr. D. Y. Patil Institute of Pharmaceutical Science and Research, Pimpri, Pune, Maharashtra 411018, India

## Abstract

In the present study bilayer patch of benzydamine HCl was developed using solvent casting method. Different substrates were attempted like Petri dish, glass-and-ring, and teflon-and-ring for selection of the proper option to formulate patch that should give easily peelable film with adequate mechanical properties. HPMC E15 LV was used in different concentrations for obtaining proper viscosity of solution for pouring on to surface and ring, that it should not leak from ring. The second layer was optimized by using different polymer like eudragit RSPO, eudragit RSPO + EC, and eudragit NE30 D for efficient layer bonding. The minimum release from backing membrane was established by diffusion study as compared to from drug loaded layer. The optimized batches were evaluated for folding endurance, weight variation, thickness, drug content, drug release, tensile strength, layer separation, mucoadhesion, moisture uptake, and layer bonding. The novel gingival patch of benzydamine HCl developed would be beneficial in optimizing the therapy.

## 1. Introduction

The concentration of drugs, when taken orally or by intramuscular route, in the blood increases and reaches its peak value in few minutes to hours. After reaching the peak blood level the drug then starts decreasing in concentration and hence must be readministered when the concentration is thought to be reduced, discussed by Kornman, Carranza [1, 2].

Pharmaceutical technologists today are able to provide drug delivery systems with very precise control over drug release for a prolonged period of time, eliminating the need for a frequent dosing and minimizing side effects, thereby increasing patient compliance and comfort. In conventional mode of administration, many drugs do not reach the target area in the body in sufficient concentration because many are prematurely inactivated and excreted. This problem can be overcome by administering the drugs directly into the intended site of action with lesser dose, discussed by Venkatesh, Jain [3, 4].

Localized drug delivery to tissues of the oral cavity has been investigated for the treatment of periodontal disease and bacterial and fungal infection, amongst the various routes in the novel drug delivery systems. Optimizing localized drug delivery mucoadhesion has potential, by retaining a dosage form at the site of action or systemic delivery. Bioadhesion is the ability of a material (synthetic or biological) to adhere to a biological tissue for a significant period of time. The biological surface can be epithelial tissue or it can be the mucus membrane over the tissue. Discussed by Raghavendra Rao, Poddar [5, 6], Nonsteroidal Anti-Inflammatory Drugs (NSAID) have been widely used for their application in dentistry which consists of analgesic and anti-inflammatory effects [6]. These drugs are used for relieving pain in periodontal disease and retarding the disease process. NSAID act by inhibiting inflammatory enzymes triggered by cytokines, which are important immune factors in periodontal disease.

A number of NSAID have been reported to reduce gingivitis and slow progression of periodontal disease, discussed by Slots and Rams [7].

Prostaglandin synthetase inhibitor benzydamine HCl is an anti-inflammatory, antimicrobial, and local anaesthetic. It is free of adverse systemic effects and is not ulcerogenic. Gingivitis involves inflammation of gums which leads to destruction and breakdown of periodontal structures resulting in increased pocket depth, clinical attachment loss, and destruction of alveolar bone. Therefore the benzydamine HCl would be an ideal choice to treat the gingival infection. Benzydamine HCl is available as mouth wash in concentration of 0.15%. The objective of the present study was to design suitable periodontal double layer patch of benzydamine HCl by using teflon as a casting substrate and HPMC E15 LV as a drug releasing polymer (first layer) and eudragit NE 30D as a backing polymer (second layer) to avoid loss of drug through back side of the patch and to evaluate for the physicochemical characteristics, including in vitro drug release.

## 2. Material and Method

### 2.1. Material

Benzydamine HCl was provided as gift sample from Alkem Laboratories Pvt. Ltd., Mumbai, India. Eudragit NE 30D was obtained as gift sample from Evonik India Pvt. Ltd. HPMC E15 LV was purchased from Colorcon Asia, Goa, India, and PEG 400 was purchased from Research Labs Finechem Industries, Mumbai, India.

### 2.2. Method

#### 2.2.1. Preparation of Double Layer (DL) Patches

Patches were prepared using the solvent casting method. The film forming polymer was dissolved by solvating overnight. Clear bubble-free solutions were poured on a film casting substrate arranged horizontally with the help of a spirit level. Pool of liquid with uniform thickness was evaporated. The dried film that resulted was peeled off.

#### 2.2.2. Casting of First Layer (FL)

The first layer was developed as a film by using teflon coated surface of an iron and stainless steel ring by the solvent casting method. For avoiding leakage the fitment of the ring and the viscosity of solution were optimized by taking different concentration of HPMC, namely, 600 mg, 650 mg, and 700 mg. The HPMC was dissolved in distilled water and IPA in ratio of 40 : 60. The PEG 400, 1 mL, was included as plasticizer. The quantity of drug was taken based on quantity in mouth wash. Mouth wash contains 22.5 mg of drug; therefore 2 mg of drug was taken to formulate patch. The resultant solution was poured on teflon coated iron surface confined by stainless steel ring. It was allowed to dry in air overnight.

#### 2.2.3. Casting of Second Layer (SL)

Second layer was cast on the surface of first layer which was obtained by confining with a ring. Eudragit NE 30D 2 mL was poured on the surface of first layer. It was allowed to dry overnight.

#### 2.2.4. Design of Experiment

 See Table 1.

#### 2.2.5. Physicochemical Properties of Drug and Polymer

Characterisation of drug and polymer was performed by using FTIR (Shimadzu-8400S) discussed by Nandgude and Bhise [8] and DSC (PerkinElmer-4000) discussed by Nandgude et al. [9].

#### 2.2.6. Evaluation of Double Layer (DL) Patches

This was discussed by Agarwal, Singh, Prabhushankar, and Beetha Rohini [10–14].

*(1) Physical Evaluation*

*(a) Thickness [11, 12].* The thickness of film was measured by digital vernier caliper Schr.Lab/DSR II (184)/1. The thickness was measured at five different sites and average was taken.

*(b) Percent Flatness [10, 11]*. The percent flatness was measured by cutting a film into strips from centre of the film. The strips were so cut that each should have had 4 cm length and 0.5 cm breadth. Each strip was put on a clean surface without applying any pressure and measured for length by digital vernier caliper Schr.Lab/DSR II (184)/1.

*(c) Moisture Uptake [10, 11].* Desiccators were used for humidity creation, one for 58% RH and another for 79% RH. Saturated solution of sodium bromide and aluminium chloride at RT (approx. 35°C) was used, respectively.

The patches were dried for 24 hours in a desiccators containing calcium chloride. Then the patches were kept in desiccators of 58% RH and 79% RH, respectively. The patches were equilibrated at respective RH for 48 hours.

*(d) Weight Uniformity [11, 12].* Five patches of 2 × 2 cm^2^ were taken from different areas of cast; each was weighed on an electronic balance and the mean weight was recorded. Uniformity in the weight of the patches was determined.

*(e) Surface pH [11, 12].* Agar plates were prepared by dissolving 2% (w/v) agar in warmed water and pouring into a Petri dish to solidify at room temperature. The patches were left to swell for 1 hour on the surface of the agar plate. The surface pH was measured by means of pH paper placed on the surface of the soaked patch.

*(2) Mechanical Properties*

*(a) Tensile Strength [12]*. The tensile strength was determined by an apparatus. Three strips of patch were cut having 4 cm length and 0.5 cm breadth. The thickness and breadth of strips were noted at three sites and average value was taken for calculation. The strips were marked with ink 2 cm apart and 1 cm from each end. Each strip was kept in the clips such that the markings were visible. The rate of change of stress was kept constant with increment. The elongation was observed and the total weight taken was used for calculation. The tensile strength was calculated using the following formula:

(1) $$\begin{array}{c} \left(\;S\;\right)=\frac{m\times g}{b\times t}, \end{array}$$

where *S* is tensile strength in dynes/cm^2^,  *m* is mass in grams, *g* is acceleration due to gravity (980 cm/sec^2^),  *b* is breadth of strip in cm, and *t* is thickness of strip in cm.

The strain undergoes change in size after a force is applied. Therefore, the strain can be given as

(2) $$\begin{array}{c} \text{Strain}\left(\;E\;\right)=\frac{L-L_{0}}{L_{0}}, \end{array}$$

where *L* is length after force was applied and *L*_0_ is original length.

*(b) Elongation [13, 14]*. The percent elongation at break was measured by the formula given below:

(3) $$\begin{array}{c} \%\text{ elongation}=\frac{L-L_{0}}{L_{0}}\times 100, \end{array}$$

where *L* is length after force was applied and *L*_0_ is original length.

*(c) Mucoadhesion Study*. The mucoadhesion study was performed by using goat gingiva. The study was performed using a texture analyser method. The sample contact time was 10 sec and load was 10 g.

*(d) Content Uniformity [11, 12, 15]*. The patch of area 4.0 cm^2^ was dissolved in 10 mL methanol and transferred to 100 mL volumetric flask, volume made up by freshly prepared phosphate buffer having pH 6.8 and kept at room temperature. After disintegration of patch, drug and polymer had got dissolved. An aliquot of 1 mL was withdrawn from the solution and diluted to 10 mL with phosphate buffer pH 6.8. The absorbance of the solution was taken at *λ*_max_ 214 nm against the solvent phosphate buffer pH 6.8 as blank.

*(e) Microscopy*. TS was prepared with a sharp blade. It was observed for the status of the two layers and their bonding under optical microscope at 100x.

*(3) Drug Release Studies*

*(a) Dissolution Studies [10, 15].* The dissolution study was performed by using USP type 1 basket type six station dissolution apparatus where previously wet patch of each batch was kept inside the basket. The dissolution medium consisted of phosphate buffer of pH 6.8 at 37 ± 1°C and 50 rpm rotation was maintained throughout the experiment. Samples were withdrawn at the intervals of 30 min. and replaced with equal volume of dissolution medium each time.

The release study was carried out for 5 hours. Withdrawn samples were filtered, diluted with phosphate buffer of pH 6.8, and then analysed spectrophotometrically at *λ*_max_ 214 nm. The patches were also noted critically for maintenance of layer bonding.

*(b) Diffusion Studies [10]*

*Diffusion Cell*. Diffusion studies are performed to obtain an idea of permeation of a drug through a barrier from a system. Usually, two types of diffusion cells are used, namely, horizontal and vertical. Franz and Keshary-Chien (K-C) type diffusion cells are of vertical type. In this work, Franz type of diffusion cell was used.

Diffusion cells generally comprise two compartments, receiver and donor compartment containing the API to dissolve and diffuse. Acceptor compartment contains a medium to receive the diffused API. The compartments are separated by barrier which was dialysis membrane.

## 3. Result and Discussion

### 3.1. Physicochemical Characterisation

#### 3.1.1. FTIR

**Figure 1:**
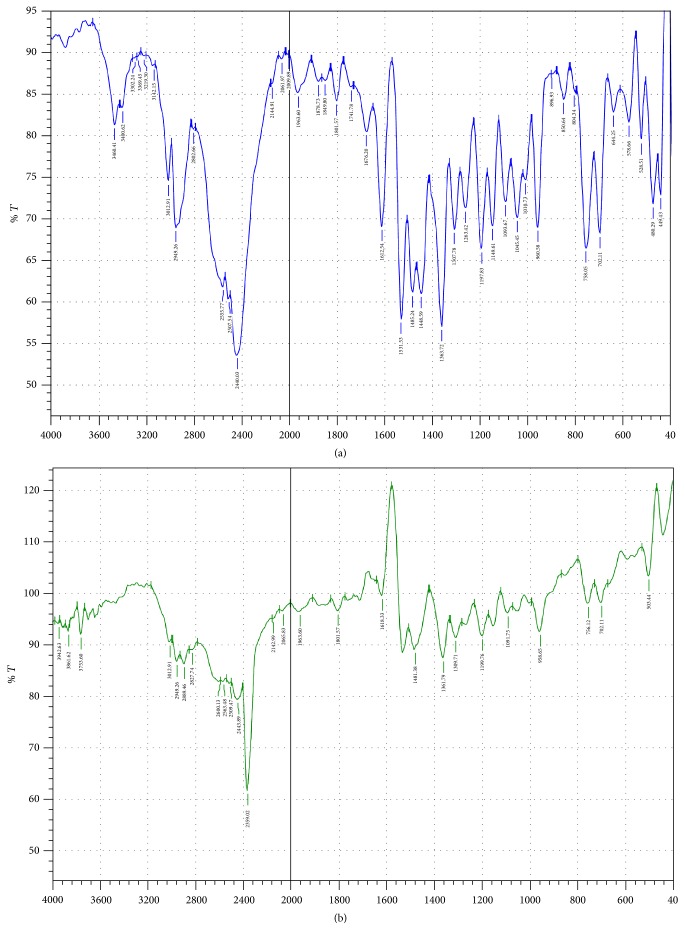
IR spectra of drug and polymer, (a) IR of benzydamine HCl, and (b) IR of physical mixture of benzydamine HCl and HPMC E15 LV.

IR spectra of drug and polymer indicated the purity of drug and polymer and absence of interaction between drug and polymer. Results are shown in Figure 1.

#### 3.1.2. DSC

**Figure 2:**
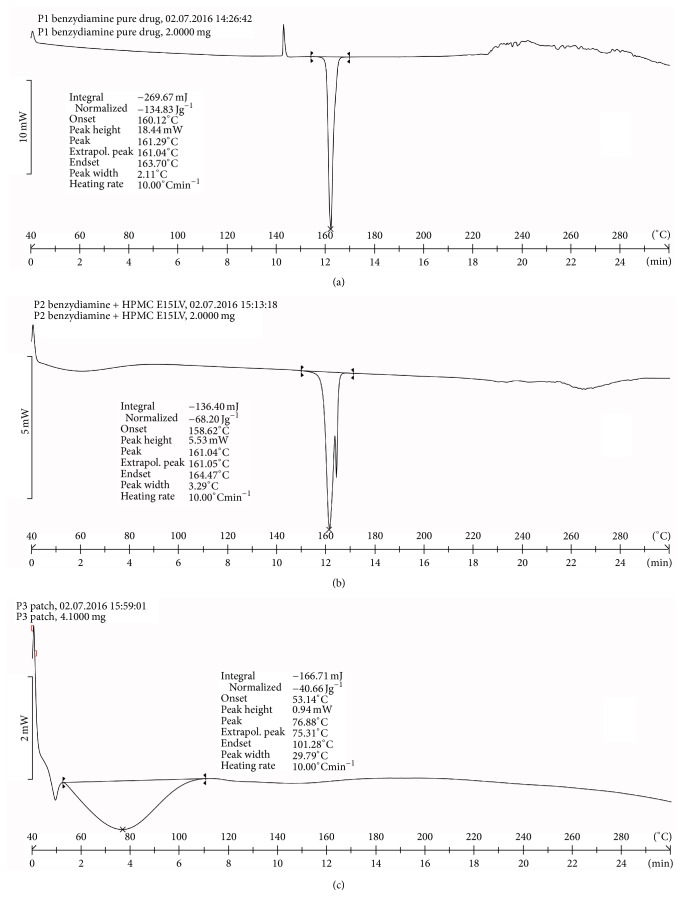
DSC patterns of (a) benzydamine HCl, (b) physical mixture of benzydamine HCl and HPMC E15 LV, and (c) patch (batch DL9).

Figure 2(a) shows endotherm of pure benzydamine HCl was found as 161.29°C. This represented the melting point for the drug.

Figure 2(b) shows the DSC graph of physical mixture of the drug and the polymer. There was no change in the endotherm drug. The melting point of drug was 161.04°C. The graph showed the compatibility of the drug and polymer.

Figure 2(c) shows the DSC graph of a formulated patch. It did not show the drug peak. This meant drug might have got changed into another form like amorphous.

### 3.2. Results of Patches of Factorial Design

#### 3.2.1. Physical and Mechanical Evaluation

The double layer patches were studied using different polymers, individually and in combinations. Eudragit RSPO gave rather brittle layer on the surface of first layer. Batches from DL4–DL6 were of combination of ethyl cellulose and eudragit RSPO with different concentration of TEC as plasticizer. The patches obtained of this combination were opaque in nature and nonuniformly spread over the first layer. Batches number DL7–DL9 contained the second layer of eudragit NE30 D. These were thin, flexible, and transparent and gave maximum percent elongation. The pH of patches from batches number DL1–DL9 was found to be in range of 6-7.

As the proportion of HPMC and PEG 400 increased in the first layer moisture uptake increased significantly. HPMC being hydrophilic in nature increased in the moisture uptake which might be due to the increase in the concentration of HPMC.

The percent moisture loss was also found to be proportional to the concentration of HPMC. In case of second layer acetone was used as solvent in batches DL1–DL6; therefore these did not show maximum percent moisture loss. This indicated that water holding was more in case of aqueous operation. Some moisture would be essential for the desirable mechanical properties. At the same time lesser moisture in the patch especially FL would make them water “thirsty.” This quality would be helpful when the patch would be applied on the gingival mucosa, helping in mucoadhesion (Table 2).

Batches DL1–DL3 gave the minimum percent elongation. This could be due to the brittle nature of second layer. The batches from DL4 to DL9 had percent elongation more as the concentration of PEG 400 and TEC was higher in them.

Batches from DL1–DL3 gave the layer separation because of the brittle nature of second layer. Second layer of these batches consisted of eudragit RSPO. As the combination of eudragit and ethyl cellulose was used it gave the weak bonding in between first and second layer, though it needed the plasticizer. Batches from DL7 to DL9 consisted of eudragit NE30 D dispersion as second-layer forming agent. There was no need of plasticizer in them as there were good plasticity and bonding of the layer.

Tensile strength was found to be changing as the use of the polymer changed. In case of eudragit RSPO it was observed that required force was low as compared to eudragit NE30 D and the combination of EC and eudragit RSPO.

As the concentration of HPMC E15 LV increased the weight also increased. This might be because of moisture retention by the HPMC E15 LV. As the concentration of HPMC increased the moisture uptake increased, and that resulted in increase in weight (Table 3).

### 3.3. Dissolution Study

**Figure 3:**
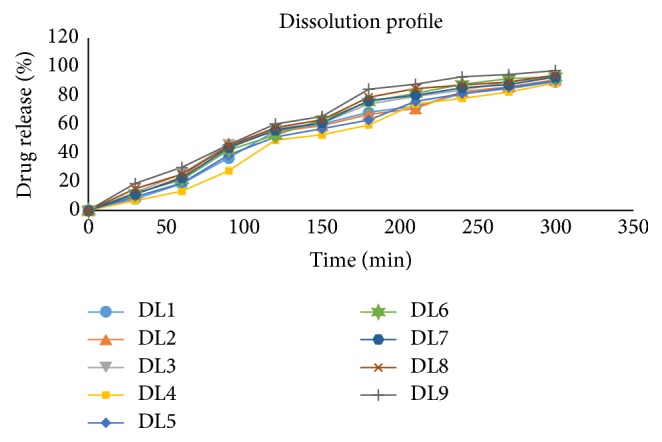
Drug release from double layer patches by dissolution study.

The dissolution study of the double layer patches is shown in Figure 3. The release of drug was in the range 88–96%. Batch DL9 gave a 96.4% drug release. During dissolution study layer separation study was executed. It was observed that there was no layer separation during and at the end of the dissolution period. This indicated significant layer bonding.

### 3.4. Diffusion Study

*(a) Front Release.* Drug release by diffusion study of front layer of double layer patch gave less diffusion as compared to the monolayer. It might be because of the presence of second layer. The release obtained was found to be in the range of 61–87% in 5 hours. The batches from DL1 to DL6 gave lesser release as compared to DL7–DL9. This indicated that as the concentration of HPMC increased release also increased. The remaining 10–15% of drug might have penetrated into the second layer.

In DL1–DL3 backward diffusion was higher due to improper layer bonding. The 10–15% of drug might have penetrated in the second layer because of dissolution of HPMC in solvent of the second layer. The nature of second layer in batches DL1–DL3 was brittle and layer separation was observed in them.

**Figure 4:**
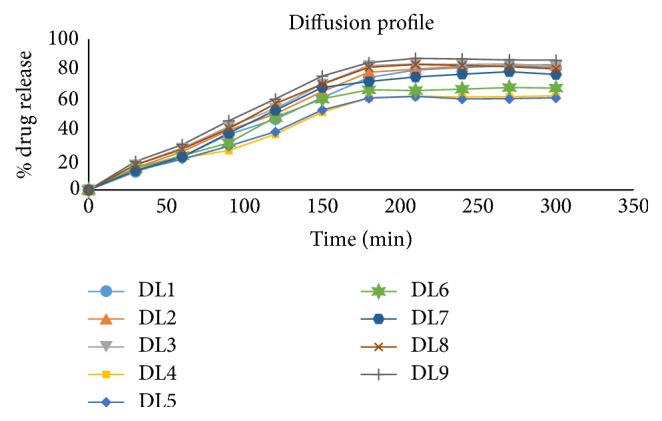
Drug diffusion from front side of double layer patches.

It was observed that there was no layer separation during and at the end of dissolution period in DL7–DL9. DL4–DL6 showed the separation of the layer, and it might be because of weak bonding in FL and SL (Figure 4).

*(b) Back Release.* It could be concluded that the 13–16% of drug diffused into and through the second layer. Batches from DL7 to DL9 gave minimum drug diffusion from second layer.

Eudragit RSPO gave back release up to 17%. The combination of eudragit RSPO and ethyl cellulose gave up to 15%. The eudragit NE30 D gave release up to 13%.

**Figure 5:**
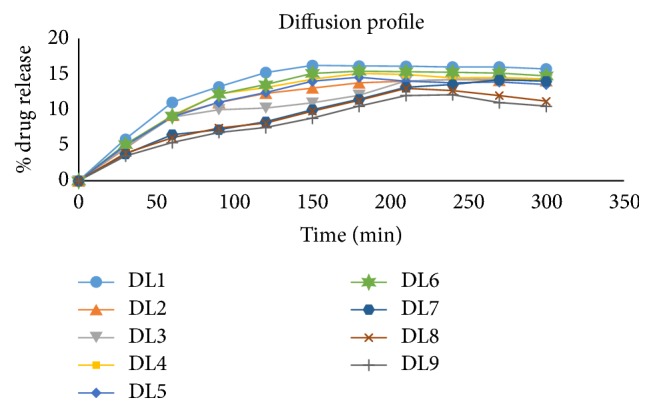
Drug diffusion from back side of double layer patches.

The eudragit NE30 D was found to have lowest permeability as compared to the eudragit RSPO and combination of eudragit RSPO and EC in case of benzydamine HCl. Therefore the batches from DL7 to DL9 were considered as the optimized batches giving the minimum release from backing layer and probably helping to have maximum release from front side (Figure 5).

**Figure 6:**
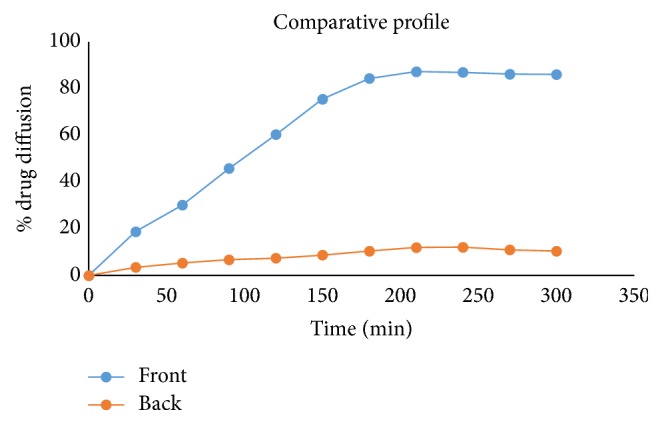
Comparative drug release from both sides of double layer patches by diffusion study.

*(c) Comparative Study*. The comparative release of drug from front and back surfaces of batch DL9 showed that up to 13% drug was released from backing layer and up to 87% of drug was released from front layer. From the above results it could be concluded that passage of drug could be minimum through the backing acrylate layer through fabrication of double layer patches (Figure 6).

### 3.5. Mucoadhesion Study

It is the ex vivo mucoadhesion with Brookfield Texture Analyser on goat gingival mucosa. Adhesion for formulation DL9 containing PEG 400 1 mL was found to be 1.9 g/cm^2^. The adhesive force generated with formulation DL9 was found to be 7.6 g (Table 4).

### 3.6. Microscopy

**Figure 7:**
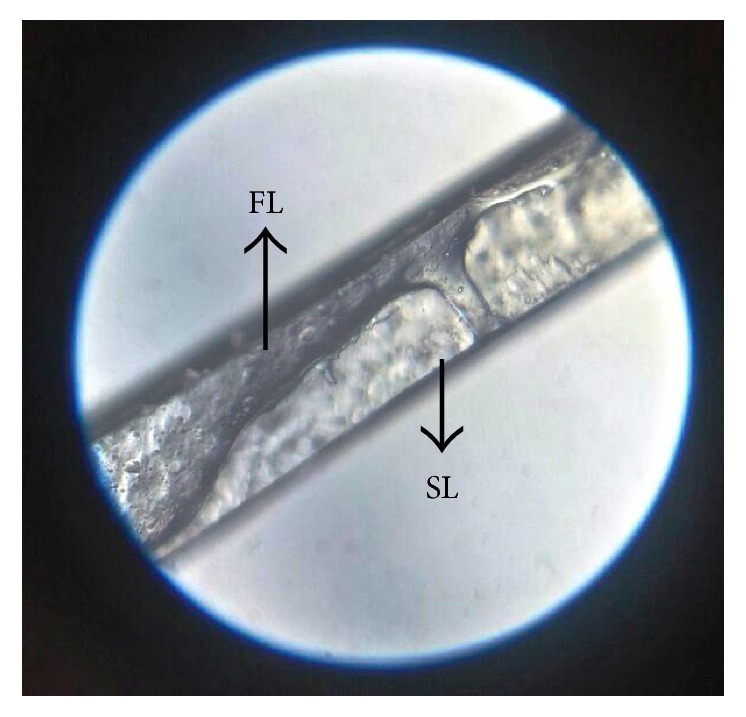
Layer bonding study by optical microscope, where FL is first layer and SL is second layer.

Layer bonding status was reported through optical microscopy at 100x, the bonding between first layer and second layer. Fine channels indicated intermixing and interpenetration of layer. This might have happened due to the addition of the second-layer solution over the dry first layer as well as during the subsequent drying. Interpenetration of layer might be due to dissolving of HPMC in second-layer liquid. Interpenetration of layers leads to good layer bonding but might lead to back release. Though it might have led to good layer bonding, it also gave a bit higher drug release through the second layer (Figure 7).

## 4. Conclusion

From the obtained results, it could be concluded that periodontal bilayered patches of benzydamine HCl were formulated by solvent casting technique using teflon coated iron and ring. The IR spectra revealed that there was no interaction between polymers and drug. All the polymers used were compatible with the drug. DSC showed purity of drug by thermogram. Evaluation parameters like thickness, tensile strength, folding endurance, and percentage moisture loss indicate that patches were mechanically stable. Percentage weight variation and content uniformity were found to be uniform in all the patches. In vitro drug release showed an abrupt release after 2 to 2.5 h. and there after the release profile was controlled and extended till the end of static release study. It also did not show layer separation of double layer patch. All the patches were found to be stable over the storage period and conditions tested. Drug release from backing membrane was minimum that would result in less loss of drug in GI track. The high % drug release of the patch indicated that these patches could be real alternative to traditional OTC product like tablets, mouth washes, gels, and capsules. This could be helpful for the treatment of gingivitis. Further investigation is required to establish in vivo and clinical efficiency of these patches.

## Figures and Tables

**Table:** (a) First Layer

Batches	Benzydamine HCl	HPMC E15 LV	PEG 400	IPA	Dist. water
	(mg)	(mg)	mL	mL	mL
DL1	32.14	600	—	12	8
DL2	32.14	650	0.5	12	8
DL3	32.14	700	1	12	8
DL4	32.14	600	—	12	8
DL5	32.14	650	0.5	12	8
DL6	32.14	700	1	12	8
DL7	32.14	600	—	12	8
DL8	32.14	650	0.5	12	8
DL9	32.14	700	1	12	8

**Table:** (b) Second layer

Batches	Eudragit RSPO	Eudragit RSPO + EC	Eudragit NE30 D	Acetone	TEC
	mg	mg	mL	mL	%
DL1	1680	—	—	20	—
DL2	1680	—	—	20	2
DL3	1680	—	—	20	3
DL4	—	1428 + 272	—	20	—
DL5	—	1428 + 272	—	20	2
DL6	—	1428 + 272	—	20	3
DL7	—	—	2	—	—
DL8	—	—	2	—	—
DL9	—	—	2	—	—

**Table 2:** Physical evaluation of double layer patches.

Batches	Appearance	Thickness (mm)	Flatness (%)	Surface pH	Moisture Loss (%)	Moisture uptake (%)	
						At 58% RH	At 79% RH
DL1	Thin, rigid, transparent	0.3 ± 0.015	90.3	6-7	1.36	2.4	5.4
DL2	Thin, rigid, transparent	0.29 ± 0.019	91.9	6-7	2.41	3.2	7.1
DL3	Thin, rigid, transparent	0.31 ± 0.019	92.1	6-7	3.54	3.8	7.5
DL4	Thin, flexible, transparent	0.28 ± 0.020	98.20	6-7	3.67	5.8	7.5
DL5	Thin, flexible, opaque	0.30 ± 0.018	96.9	6-7	3.20	6.2	7.8
DL6	Thin, flexible, transparent	0.32 ± 0.13	95	6-7	4.26	6.5	4.3
DL7	Thin, flexible, transparent	0.27 ± 0.16	98.45	6-7	4.35	3.4	7.1
DL8	Thin, flexible, transparent	0.30 ± 0.11	97.94	6-7	3.98	4.6	5.1
DL9	Thin, flexible, transparent	0.25 ± 0.21	98.93	6-7	3.55	5.4	6.4

**Table 3:** Mechanical evaluation of double layer patches.

Batches	Tensile strength (dynes/cm^2^)	Elongation (%)	Weight (mg)	Folding endurance		Drug content (%)
				Layer separation	Yield number of folds	
DL1	33.7 × 10^6^ ± 21.7	46 ± 0.8	105	Yes	>120	94.21
DL2	36.9 × 10^6^ ± 1.7	44 ± 0.8	108	Yes	>125	94.70
DL3	37.5 × 10^6^ ± 1.7	48 ± 0.8	110	Yes	>130	95.01
DL4	39.8 × 10^6^ ± 2.0	52 ± 1.8	106	No	>125	95.32
DL5	42.1 × 10^6^ ± 2.0	55 ± 1.8	109	No	>135	94.65
DL6	43.14 × 10^6^ ± 2.0	58 ± 1.8	112	No	>145	96.2
DL7	45.02 × 10^6^ ± 1.5	56 ± 1.8	111	No	>150	95.11
DL8	44.1 × 10^6^ ± 1.5	59 ± 1.8	114	No	>150	96.98
DL9	46.26 × 10^6^ ± 1.5	60 ± 1.8	115	No	>160	98.5

**Table 4:** In vitro mucoadhesion strength measurement with Brookfield Texture Analyser.

Batch	Adhesive force	Adhesiveness
DL9	7.6 g	1.9 g/cm^2^
